# Vesicocutaneous Fistula Following Radiation and Surgery for the Treatment of Rectal Cancer

**DOI:** 10.7759/cureus.16219

**Published:** 2021-07-06

**Authors:** Mustafa Ridha, Conor Honeywill, Jason Diab, Sarit Badiani, Christophe R Berney

**Affiliations:** 1 Surgery, Bankstown-Lidcombe Hospital, Sydney, AUS; 2 Surgery, New South Wales (NSW) Health, Sydney, AUS; 3 Surgery, University of New South Wales, Sydney, AUS; 4 Surgery, University of Notre Dame, School of Medicine, Sydney, AUS

**Keywords:** vesicocutaneous, fistula, radiotherapy, rectal cancer, cutaneous metastasis

## Abstract

Vesicocutaneous fistulas (VCF) are abnormal tracts formed between the bladder and the cutaneous surfaces of the body. Although rare, it has been reported following radiotherapy to the pelvic region, surgery and trauma. We present a case of a 70-year-old male who underwent neoadjuvant chemoradiotherapy for the treatment of rectal cancer metastatic to the perineum prior to definitive abdominoperineal resection (APR). Six months later, he developed urinary retention secondary to bladder obstructive outlet disease. This was managed with urinary catheterisation and a month later with transurethral resection of the prostate (TURP). At outpatient follow-up, he complained of urinary leakage in the perineal region approximately 10 months post-chemoradiotherapy. He underwent a computer tomography (CT) cystogram which confirmed the findings of a VCF extending to his perineum scar. He was managed conservatively with successful outcomes using a multidisciplinary team approach. This is the first case of delayed VCF reported arising after chemoradiotherapy for locally advanced rectal colorectal cancer.

## Introduction

A vesicocutaneous fistula (VCF) is a rare phenomenon where an abnormal tract forms between the bladder and the cutaneous surface of the body. Risk factors include trauma, bladder calculi, surgery and radiotherapy to the pelvic region [1-3]. The onset of symptoms is variable occurring immediately or up to 30 years later [4]. We present a case of a VCF in a 70-year-old male following a transurethral resection of his prostate (TURP) with a background of recent neoadjuvant chemoradiation therapy and surgery for locally advanced rectal cancer.

## Case presentation

**Figure 1:**
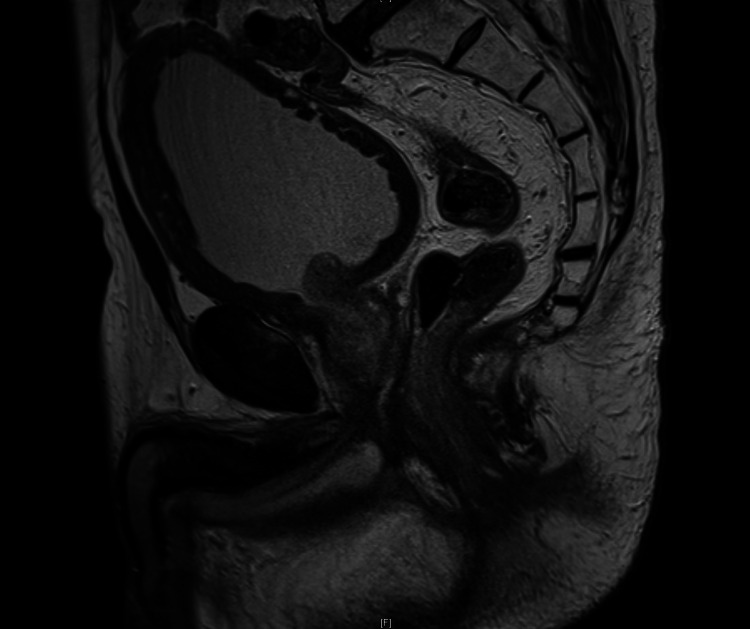
MRI Rectum Sagittal T2 section of an MRI rectum post-chemoradiation and prior to the APR. It demonstrates multiple small diverticula in the bladder wall and features of trabeculation.

**Figure 2:**
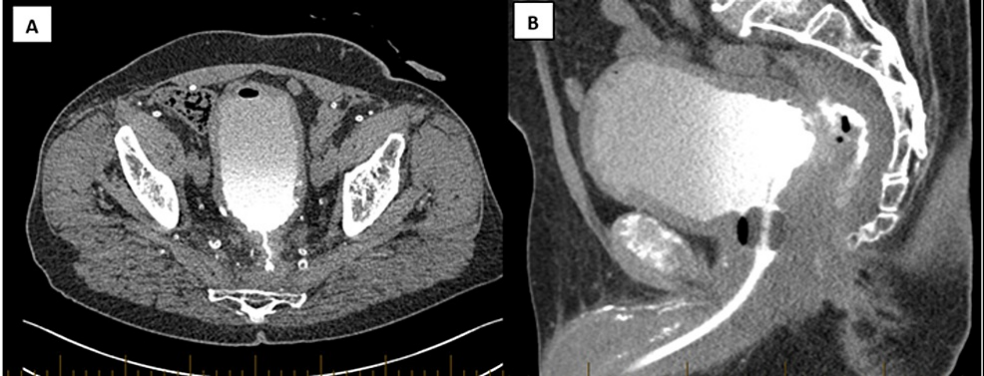
CT Cystogram (A) CT cystogram demonstrating a VCF arising from the posterior aspect of the bladder wall. It traverses the presacral space posteriorly and then inferiorly towards the perineum. (B) CT cystogram demonstrating the VCF arising from the mid-line of the posterior bladder wall with parietal irregularity.

A 70-year-old man was diagnosed with rectal cancer and a single metastatic subcutaneous perineal deposit. He was treated with a five-week course of neoadjuvant chemoradiotherapy consisting of 50 Gy in 25 fractions, with the radiation field encompassing the perineal skin, followed by an abdominoperineal resection (APR) ten weeks later without any immediate surgical complications (Figure 1). Six months later, he was reviewed in our urology outpatient clinic with a three-month history of intermittent urinary retention and clinical evidence of severe urinary retention complicated by bilateral hydronephrosis. This was initially treated by immediate IDC insertion followed by a TURP one month later. The post-surgical outcome was uneventful, however, two months later he developed worsening urinary leakage and discomfort arising from his perineum scar. A computed tomography (CT) cystogram showed a fistula tract arising from a bladder diverticulum extending towards the presacral space and inferiorly towards the perineum (Figure 2). He was managed conservatively with regular dressing changes for six weeks and the fistula tract spontaneously dried out. 

## Discussion

The development of VCF is often implicated by risk factors such as radiotherapy, surgery and trauma. Radiotherapy results in progressive microvascular injury, stromal fibrosis and impaired wound healing [5]. This subsequently leads to a decrease in bladder wall compliance, mucosal erosions with potential perforation and fistula formation [6]. Urological complications following radiotherapy for rectal cancer may also occur due to the greater radiosensitivity of the bladder compared to that of the colon and rectum. In our case, previous chemoradiotherapy with the radiation field encompassing a significant amount of the perineal skin due to metastatic deposit and repeated episodes of urinary retention are likely to have contributed to poor bladder compliance leading to progressive bladder intramural ischemia which may have been exacerbated by the TURP [7].

VCF can be managed conservatively or surgically depending on the patient’s comorbidities and clinical presentation. Conservative measures include IDC insertion and percutaneous nephrostomies to allow adequate healing of the tract [8]. Ureteric embolization can also be utilised with percutaneous nephrostomies if treatment fails or in the situation of poor life expectancy [9]. This approach may be more favourable in the setting of radiation-induced VCF as the fibrosis and overall tissue viability may increase the likelihood of further complications. Conversely, surgical measures include tissue interposition with either omentum or a femoral gracilis flap [10].

## Conclusions

To the best of our knowledge, there are currently no reported cases of VCF arising after neoadjuvant chemoradiotherapy for locally advanced rectal cancer. A multidisciplinary team involving colorectal, urology and plastic surgeons is recommended if conservative measures fail. As discussed, there are multiple options to be considered in managing and treating the condition. These should be discussed with the patient and the decision made should be based on the patient’s comorbidities and expected outcomes.
